# Metabolomics and molecular marker analysis to explore pepper (*Capsicum* sp.) biodiversity

**DOI:** 10.1007/s11306-012-0432-6

**Published:** 2012-06-02

**Authors:** Yuni Wahyuni, Ana-Rosa Ballester, Yury Tikunov, Ric C. H. de Vos, Koen T. B. Pelgrom, Awang Maharijaya, Enny Sudarmonowati, Raoul J. Bino, Arnaud G. Bovy

**Affiliations:** 1 Wageningen UR Plant Breeding, 6708 PB Wageningen, The Netherlands; 2 Research Centre for Biotechnology, Indonesian Institute of Sciences, Jl. Raya Bogor KM. 46, Cibinong, Bogor, 16910 Indonesia; 3 Centre for Biosystems Genomics, 6700 PB Wageningen, The Netherlands; 4 Plant Research International, 6700 AA Wageningen, The Netherlands; 5 Laboratory of Plant Physiology, Wageningen University, 6700 AR Wageningen, The Netherlands; 6 Bogor Agricultural University, Jl. Raya Darmaga, 16680 Bogor, Indonesia; 7 Netherlands Metabolomics Centre, Einsteinweg 55, 2333 CC Leiden, The Netherlands; 8 Present Address: Instituto de Agroquímica y Tecnología de Alimentos. Consejo Superior de Investigaciones Científicas (IATA-CSIC), Avenida Agustín Escardino 7, 46980 Paterna, Valencia Spain

**Keywords:** *Capsicum*, Metabolite profiling, Semi-polar compounds, Volatiles, Pungency, AFLP

## Abstract

An overview of the metabolic diversity in ripe fruits of a collection of 32 diverse pepper (*Capsicum* sp.) accessions was obtained by measuring the composition of both semi-polar and volatile metabolites in fruit pericarp, using untargeted LC–MS and headspace GC–MS platforms, respectively. Accessions represented *C. annuum*, *C. chinense*, *C. frutescens* and *C. baccatum* species, which were selected based on variation in morphological characters, pungency and geographic origin. Genotypic analysis using AFLP markers confirmed the phylogenetic clustering of accessions according to *Capsicum* species and separated *C. baccatum* from the *C. annuum*–*C. chinense*–*C. frutescens* complex. Species-specific clustering was also observed when accessions were grouped based on their semi-polar metabolite profiles. In total 88 semi-polar metabolites could be putatively identified. A large proportion of these metabolites represented conjugates of the main pepper flavonoids (quercetin, apigenin and luteolin) decorated with different sugar groups at different positions along the aglycone. In addition, a large group of acyclic diterpenoid glycosides, called capsianosides, was found to be highly abundant in all *C. annuum* genotypes. In contrast to the variation in semi-polar metabolites, the variation in volatiles corresponded well to the differences in pungency between the accessions. This was particularly true for branched fatty acid esters present in pungent accessions, which may reflect the activity through the acyl branch of the metabolic pathway leading to capsaicinoids. In addition, large genetic variation was observed for many well-established pepper aroma compounds. These profiling data can be used in breeding programs aimed at improving metabolite-based quality traits such as flavour and health-related metabolites in pepper fruits.

**Electronic supplementary material:**

The online version of this article (doi:10.1007/s11306-012-0432-6) contains supplementary material, which is available to authorized users.

## Introduction

Pepper (*Capsicum* spp.) is one of the most important fruit crops worldwide. It is cultivated all over the world, primarily in the tropical and subtropical countries. Pepper fruit production reached up to 1.8 million HA with more than 28 million metric tonnes harvested from the crop (FAO 2011). The fruits are mainly consumed as a fresh or cooked vegetable, a condiment or a spice and used as a colouring agent in the food industry. Pepper is increasingly recognized as an excellent source of health-related metabolites, such as ascorbic acid (vitamin C), carotenoids (provitamin A), tocopherols (vitamin E), flavonoids and capsaicinoids (Howard and Wildman 2007). We recently showed that different *Capsicum* accessions display a large variation in morphological characters as well as in the levels and composition of the above-mentioned health related metabolites (Wahyuni et al. 2011). The accessions belong to four widely cultivated species: *Capsicum annuum*, *Capsicum chinense*, *Capsicum baccatum*, and *Capsicum frutescens*. These four species can be intercrossed and produce fertile hybrids (Pickersgill 1997). Variation in morphological characters included fruit features, such as colour, type, shape and size. Fruit colour in the accessions varied from red, orange and yellow to brown and salmon and this was determined by the accumulation of specific carotenoids, i.e. capsanthin and capsanthin-esters for red accessions, violaxanthin and violaxanthin-esters for yellow accession. Fruit spiciness or pungency is determined by total levels of capsaicinoids, which varied strongly among the accessions analyzed (Wahyuni et al. 2011). Based on the range of capsaicinoid levels the accessions can be categorized into non-pungent, low, mild and highly pungent (Howard and Wildman 2007).

While the targeted metabolite analyses described above focused on the analyses of specific groups of known pepper compounds, untargeted metabolomics approaches based on mass spectrometry (MS) allow the simultaneous detection of metabolites in a biological sample, without *a priori* knowledge of the identity of the metabolites detected. Such untargeted profiling approaches have been used to obtain an overview of the metabolic diversity in germplasm collections, such as Arabidopsis (Keurentjes et al. 2006) and tomato (Tikunov et al. 2005). These analyses may help to understand metabolic pathways underlying the main metabolic contrasts between genotypes and, in combination with genetic analyses, to identify mQTLs and genes underlying key steps in metabolic pathways. In addition, metabolic profiling allows the detection and subsequent identification of unknown compounds correlating with a trait of interest, such as pungency, colour or flavour. There is no doubt that the concept of metabolomics-assisted breeding is a novel and powerful approach leading to new targets for breeding programs aimed at the improvement of metabolite-based quality traits (Fernie and Schauer 2009).

In addition to phenotypic information, knowledge about genetic diversity among accessions is also important to maximize the rate of crop improvement (Geleta et al. 2005). Genetic diversity can be measured using molecular markers and one of the methods is amplified fragment length polymorphism (AFLP) developed by Vos et al. (1995), which has been used extensively for genetic mapping and fingerprinting in plants. Combining genetic and phenotypic information, such as metabolite profile, may help breeders to develop new varieties with desired specific metabolite compositions and related traits.

Here we present a broad overview of the metabolic variation in the fruits of a collection of 32 diverse pepper accessions, by measuring the composition of semi-polar and volatile metabolites using LC–MS and headspace GC–MS, respectively. Our results showed that the diversity of volatile and semi-polar metabolites detected in the accessions separated them according to species and pungency, which correlated well with the phylogenetic relationships measured using AFLP molecular markers. Metabolite identification revealed the metabolic pathways underlying the metabolic differences between genotypes, including several known flavour-related pathways. This result offers both new sources and novel traits for the genetic improvement of the quality of cultivated pepper.

## Materials and methods

### Plant materials and sampling protocol

A total of 32 accessions from *Capsicum annuum*, *Capsicum chinense*, *Capsicum baccatum* and *Capsicum frutescens* were selected based on the variations in morphological characters and geographical origins as presented in Table 1 (Wahyuni et al. 2011). Plant growth condition, experimental design and sampling protocol were performed as described by Wahyuni et al. (2011). Briefly, the experimental design consisted of two randomized blocks, each comprising all 32 accessions grown in plots of four plants. Fruits of two biological replicates, consisting of 10–50 ripe fruits (depending on fruit size) from the two innermost plants of a plot, were harvested for each accession. Before harvesting fruits for metabolic analyses, we carefully followed the ripening stages of the first set of fruits produced by each accession. Fruit ripeness was indicated by a combination of (i) its mature colour based on the description in the CGN database, (ii) fruit firmness and (iii) number of days after fruit setting (on average 70–80 days after fruit setting). Young leaves and fruit pericarp tissue (separated from placenta plus seeds) were collected, frozen in liquid nitrogen, ground and stored at −80 °C prior to analysis.

**Table 1:** Description of 32 *Capsicum* accessions

Accession	Species	Accession name	Population type	Fruit type	Pungencyª
1	*C. annuum*	I2 Tit super	n.d.	Pointed	Low pungent
2	*C. chinense*	I1 PI 281428	n.d.	Roundish	Mildly pungent
3	*C. chinense*	I1 PI 315023 (Mishme Black)	n.d.	Conical	Mildly pungent
4	*C. annuum*	Laris HS	Breeders variety	Pointed	Low pungent
5	*C. annuum*	Jatilaba	Breeders variety	Pointed	Low pungent
6	*C. annuum*	Bruinsma Wonder	Breeders variety	Blocky/bell	Low pungent
7	*C. annuum*	PBC 473 - none - cayenne	n.d.	Pointed	Low pungent
8	*C. annuum*	PBC 535 - IR - 12 × 1 cm - cayenne	n.d.	Pointed	Low pungent
9	*C. annuum*	Bisbas	Land variety	Other	Mildly pungent
10	*C. annuum*	California Wonder 300	Breeders variety	Blocky/bell	Non pungent
11	*C. annuum*	Keystone Resistant Giant	Breeders variety	Blocky/bell	Non pungent
12	*C. annuum*	Long Sweet	Land variety	Pointed	Non pungent
13	*C. annuum*	Sweet Banana	Breeders variety	Pointed	Non pungent
14	*C. annuum*	Yolo Wonder L	Breeders variety	Blocky/bell	Non pungent
15	*C. baccatum var. baccatum*	No. 1553	Wild variety	Pointed	Highly pungent
16	*C. chinense*	Miscucho colorado; PI 152225; 1SCA no.6	n.d.	Conical	Mildly pungent
17	*C. chinense*	No. 4661; PI 159236	n.d.	Pointed	Low pungent
18	*C. chinense*	No. 4661 Selection; PI 159236 Selection	Research material	Pointed	Low pungent
19	*C. annuum*	AC 1979	Wild variety	Conical	Highly pungent
20	*C. annuum*	CM 331; Criollos de Morelos	Land variety	Conical	Non pungent
21	*C. annuum*	Sweet Chocolate	Breeders variety	Roundish	Non pungent
22	*C. annuum*	Chili Serrano; PI 281367; No. 999	Land variety	Pointed	Mildly pungent
23	*C. annuum*	Chili de Arbol; PI 281370; No. 1184	Land variety	Other	Mildly pungent
24	*C. chinense*	AC 2212	n.d.	Pointed	Low pungent
25	*C. chinense*	No.1720; PI 281426; 1GAA	Land variety	Pointed	Mildly pungent
26	*C. chinense*	RU 72-194	Land variety	Roundish	Mildly pungent
27	*C. chinense*	RU 72-241	Land variety	Other	Mildly pungent
28	*C. frutescens*	Lombok	Land variety	Pointed	Highly pungent
29	*C. frutescens*	Tabasco	Breeders variety	Pointed	Highly pungent
30	*C. baccatum var. pendulum*	Aji Blanco Christal; CAP 333	Breeders variety	Pointed	Low pungent
31	*C. baccatum var. pendulum*		n.d.	Pointed	Mildly pungent
32	*C. baccatum var. pendulum*	RU 72-51	Land variety	Other	Low pungent

^a^Denotes that the information is derived from the previous report of Wahyuni et al. (2011)

*n.d.* data not available

### Genomic DNA extraction and AFLP analysis

Genomic DNA from young pepper leaves was extracted by using the automated protocol of AGOWA^®^ mag Maxi DNA isolation kit and KingFisher 96 instrument (LGC Genomics). AFLP analysis was performed according to the protocol previously described by Vos et al. (1995). In brief, 250 ng of genomic DNA was used for restriction/ligation reaction with two restriction enzymes *Eco*RI and *Mse*I. In order to selectively amplify a smaller number of genomic DNA fragments, a pre-amplification was performed using two primers: E01 (5′-GAC TGC GTA CGA ATT CA-3′) and M02 (5′-GAT GAG TCC TGA GTA AC-3′). Amplification primers were selected as followed: E32 (E01 + AAC), E35 (E01 + ACA), E36 (E01 + ACC), E40 (E01 + AGC), M47 (M02 + CAA), M49 (M02 + CAG), M59 (M02 + CTA) and M61 (M02 + GCG). Primers E32, E36 and E40 were labeled with fluorescent IRD700 and primer E35 was labeled with fluorescent IRD 800 dye (LI-COR^®^, Lincoln, USA). DNA fragments were amplified using five primer combinations of E40–M49, E36–M47, E35–M61, E35–M59 and E32–M49. AFLP products were separated and visualized on 6.5 % denaturing polyacrylamide gel using LI-COR 4200 Global System^®^ sequencer. AFLP bands were scored based on the presence or absence of amplified DNA fragments on gel by Quantar software (Keygene^®^). Genetic distance was calculated and constructed using unweighted pair group mean average (UPGMA) and Jaccard similarity coefficient in Genemath XT version 1.6.1 software (www.applied-math.com). The reliability of the hierarchical cluster analysis (HCA) was tested using bootstrap analysis with 100 replications.

### Extraction and analysis of semi-polar metabolites

Semi polar metabolites were extracted using the protocol described previously by de Vos et al. (2007). Briefly, 500 mg of freeze-ground material of pepper pericarp were extracted with 1.5 ml of 99.875 % methanol acidified with 0.125 % formic acid. The extracts were sonicated for 15 min and filtered through a 0.2 μm polytetrafluoroethylene (PTFE) filter. For each accession two biological replicates were prepared, resulting in a total of 64 extracts. To check the technical variation, including extraction, sample analysis and data-processing, quality control samples were prepared by pooling fruit material of several randomly chosen accessions, extracted using the same procedure and injected after every 16 accession sample extracts.

All the extracts were analysed using reversed phase liquid chromatography coupled to a photodiode array detector and a quadrupole time of flight high-resolution mass spectrometry (LC–PDA–QTOF–MS) system, using C18-reversed phase chromatography and negative electrospray ionization, as described previously (de Vos et al. 2007). For LC–PDA–QTOF–MS, 5 μl of the extract were injected and separated using a binary gradient of ultrapure water (A) and acetonitrile (B), both acidified with 0.1 % formic acid, with a flow rate of 0.19 ml/min. The initial solvent composition consisted of 95 % of A and 5 % of B; increased linearly to 35 % A and 65 % B in 45 min and maintained for 2 min. The column was washed with 25 % A and 75 % B for 5 min and equilibrated to 95 % A and 5 % B for 2 min before the next injection.

The putative identification of differential semi-polar metabolites was performed by re-injection of the extract from one of the biological replicates of each accession on a LC–LTQ–Orbitrap FTMS hybrid mass spectrometer (Thermo Fisher Scientific) and performing MS^n^ fragmentation The LTQ–Orbitrap hybrid mass spectrometer system was set up in negative ionization mode, as previously described by van der Hooft et al. (2011). Xcalibur software (Thermo Fisher Scientific) was used to control all instruments and for data acquisition and data analysis.

### Extraction and analysis of volatile metabolites

Volatile metabolites were extracted using the protocol previously described by Tikunov et al. (2005). Briefly, 750 mg of freeze-ground pepper pericarp of each sample was weighed in a 5-ml screw-cap vial, closed, and incubated at 30 °C for 10 min in a water bath. An aqueous 0.75 ml of EDTA–NaOH solution (100 mM EDTA solution pH adjusted to 7.5 with NaOH) was added to the incubated sample and followed by the addition of solid CaCl_2_ (5 M final concentration). The closed vials were sonicated for 5 min and 1 ml of the extract was transferred into a 10-ml crimp vial (Waters), capped, and used directly for headspace SPME–GC–MS analysis as described in Tikunov et al. (2005). As for LC–MS, quality control samples were prepared after eight accession samples to analyse the analytical variation. Each accession was analysed with two biological replicates.

### Metabolite data processing

Both volatile and semi-polar data were processed separately through some steps as described in several points below:

#### Mass spectral alignment, filtering and clustering

Volatile and semi-polar metabolite profiles derived using the SPME–GC–MS and the LC–PDA–QTOF–MS platforms, respectively, were processed as described by Tikunov et al. (2005, 2010). Both datasets were processed independently by the MetAlign software package (www.metalign.nl) for baseline correction, noise estimation, and ion-wise mass spectral alignment. The MetAlign outputs for both the GC–MS and LC–MS data were processed separately with MSClust software for data reduction and compounds mass spectra extraction (Tikunov et al. 2011).

#### Putative identification of semi-polar and volatile metabolites

The identification of semi-polar metabolites was carried out by means of their UV spectra, exact molecular weight and MS^n^ fragmentation pattern. Putative identification of semi-polar metabolites was obtained using different metabolite databases such as Dictionary of Natural Products (http://dnp.chemnetbase.com), KNApSAcK (http://kanaya.naist.jp/KNApSAcK) and in-house metabolite databases, and using previous results on pepper described by Marin et al. (2004) and Wahyuni et al. (2011).

Putative identification of volatile metabolites was performed by automatic matching of their mass spectra extracted by MSClust with the National Institute of Standards and Technology (NIST) mass spectral library entries, using the NIST MS Search v2.0 software (http://chemdata.nist.gov/mass-spc/ms-search/). The compound hit that showed the highest matching factor (MF) value (≥600) and the lowest deviation from the retention index (RI) value was used for the putative metabolite identity. Additional manual spectral matching was performed for selected metabolites with low MF and high RI deviations by deconvoluting the chromatographic peak at the corresponding retention time using AMDIS (version 2.64) (http://www.amdis.net/) followed by matching the resulting spectra with those in the NIST library database.

#### Multivariate analysis

Semi-polar and volatile metabolite data sets containing the intensity levels of all centrotypes for all pepper samples were analysed separately using multivariate statistical analyses included at the Genemath XT version 1.6.1 software. Pre-treatment of the data was performed by log_2_ transformation and mean centering. The pre-treatment data were subjected to analysis of variance (ANOVA) and multiple testing error rates (Bonferroni procedure) to determine the least significance variances among pepper accessions. Metabolites that showed significant differences between accessions, determined by *p*-values lower than 0.001, were subjected to principal component analysis (PCA) and hierarchical cluster analysis (HCA). HCA was performed by using the UPGMA method and Pearson’s coefficient matrix in Genemath XT software. To test the reliability of the dendrogram produced by HCA, bootstrap analysis was performed with 100 replications.

## Results

### Genetic diversity of *Capsicum* accessions based on AFLP analysis

**Fig. 1:**
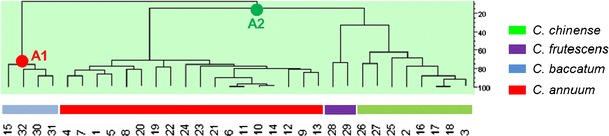
Dendrogram resulting from a hierarchal cluster analysis (HCA) based on the distribution patterns of the AFLP markers. The numbers at the bottom of the figure correspond to the accession numbers shown in the first column of Table 1

The collection of 32 pepper accessions derived from diverse origins (Table 1). To get insight into the genetic relationships, all plants were genotyped using AFLP analysis. Screening of the accessions with five *Eco*RI × *Mse*I primer combinations resulted in the identification of 255 polymorphic bands. A dendrogram resulting from HCA was constructed by counting the presence or absence of these polymorphic bands. The dendrogram showed that the 32 accessions were separated based on *Capsicum* species (Fig. 1). Two main clusters were observed, differentiating between *C. baccatum*, denoted as A-1, and the *C. annuum*–*C. frutescens*–*C. chinense* cluster, denoted as A-2. Cluster A-1 grouped all *C. baccatum* accessions analysed, including *C. baccatum var. baccatum* (no. 15) and three accessions of *C. baccatum var. pendulum*: Aji Blanco Christal (no. 30), no. 31, and RU 72–51 (no. 32). Cluster A-2 contained two sub-clusters that distinguished all *C. annuum* accessions from *C. frutescens*–*C. chinense*.

In the *C. frutescens*–*C. chinense* sub-cluster, the two *C. frutescens* accessions, Lombok (no. 28) and Tabasco (no. 29) clustered together and were separated from the *C. chinense* cluster. Within the *C. annuum* sub-cluster, several subgroups could be observed, e.g. accessions with pointed fruits (accession no. 4, 7, 1, 5, 8, and 20) and the blocky-type breeding varieties (accession no. 21, 6, 11, 10 and 14). In addition, we observed that accession *C. chinense* AC 2212 (no. 24) was positioned in the *C. annuum* cluster, suggesting that this accession, genetically, more likely belongs to *C. annuum* than to *C. chinense*. Based on this dendrogram, *C. chinense* AC2212 (no. 24) is denoted as *C. annuum* for further discussions.

### Principal component and hierarchical cluster analyses of *Capsicum* accessions based on their metabolites profiles

In order to explore the metabolic variation in pepper fruits, two different analytical platforms were used: LC–PDA–QTOF–MS for the detection of semi-polar metabolites, such as alkaloids, flavonoids and other phenolic compounds, and headspace GC–MS to detect flavour-related volatiles.

#### Semi-polar metabolites

Pericarp extracts from ripe fruits of the 32 pepper accessions were analysed by LC–PDA–QTOF–MS and the corresponding chromatograms were subjected to full mass spectral alignment using MetAlign software followed by filtering out of low intensity ions. Using the MSClust algorithm, a total of 11,372 ions were grouped into 881 ion clusters, representing reconstructed metabolites. From each mass cluster, the most abundant ion was selected as the representative of each putative compound and subsequently used for analysis of variance. In total 297 compounds showed significant intensity differences (ANOVA; *p* < 0.001) between *Capsicum* accessions. These were used for further analyses, supplemented with 34 additional metabolites, which did not meet the above criteria, but for which a putative identification was obtained (Supplementary Table S1).

**Fig. 2:**
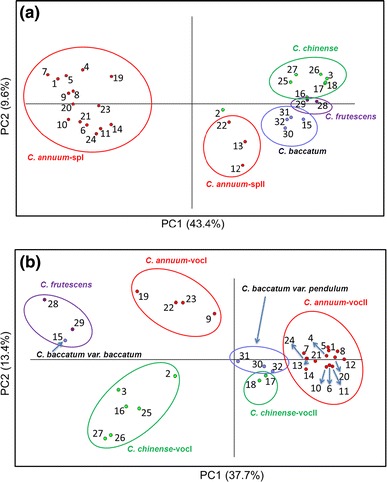
Principal component analysis (PCA) of pepper accessions based on semi-polar (**a**) and volatile (**b**) metabolite profiles. The numbers in PCA correspond to the accession numbers as shown in the first column of Table 1

PCA revealed a separation of the 32 pepper accessions into species groups, based on their semi-polar metabolite patterns (Fig. 2a). The first principal component (PC1) explained 43.4 % of the variation and separated a group of 15 *C. annuum* accessions, denoted as *C. annuum*-spI, from the other 17 *Capsicum* accessions. The second principal component (PC2) explained 9.6 % of the variation and separated the *C. chinense* group from *C. baccatum*, *C. frutescens* and the sub-group of *C. annuum*, denoted as *C. annuum*-spII. The latter group contained *C. annuum* Long Sweet (no. 12), *C. annuum* Sweet Banana (no. 13) and *C. annuum* Chili Serrano (no. 22).

**Fig. 3:**
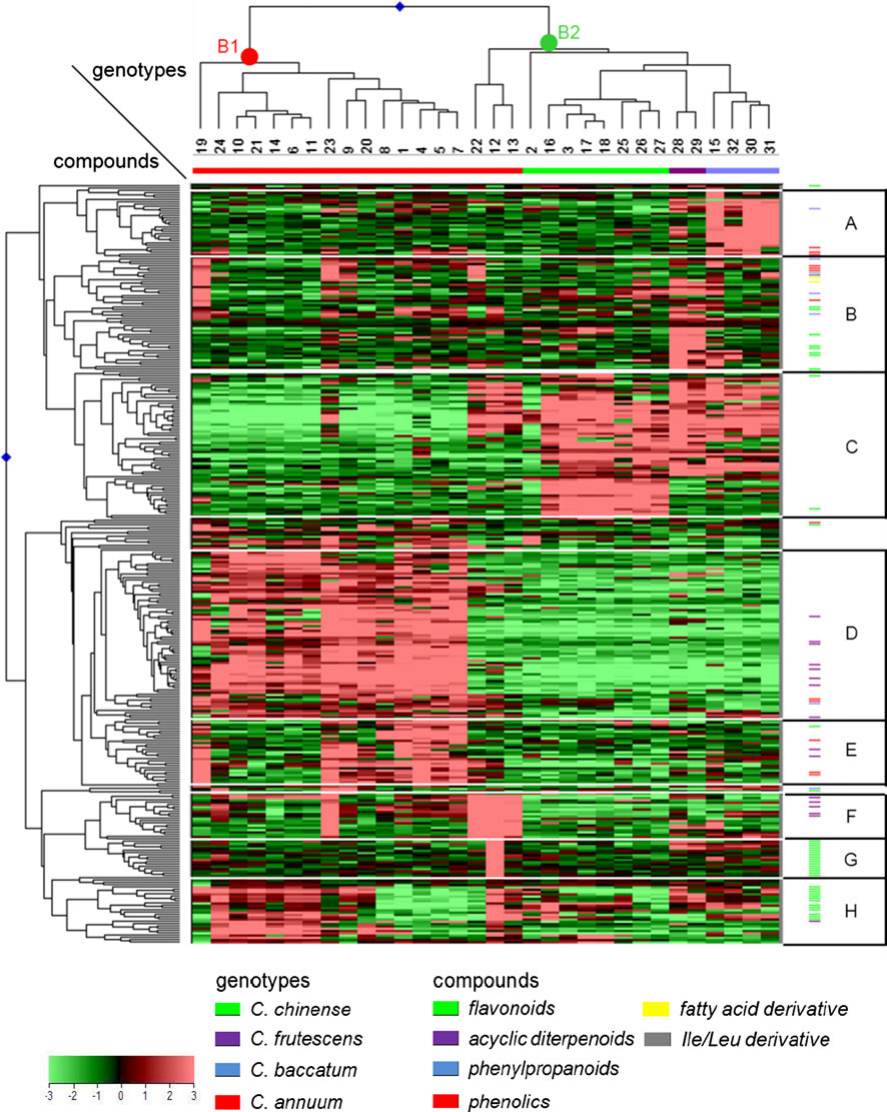
Heat map of 331 semi-polar metabolites in 32 pepper accessions. A *color-coded* matrix represents the mean values of the metabolite intensity in two biological replicates of pepper accessions, which has been log_2_ transformed and mean-centered. The *alphabets* (*A–H*) represent metabolite clusters. Characteristics of the underlying metabolites are presented in the Supplemental Table S1. The numbers below the dendrogram correspond to the accession numbers in the first column of Table 1

The dendrogram produced by HCA of the pepper accessions, based on the variation in 331 semi-polar metabolites (Fig. 3), confirmed the results observed with PCA and provided a more detailed view on the relationships between accessions. HCA distinguished two major clusters, denoted as B-1 and B-2. Cluster B-1 consisted of the *C. annuum*-spI accessions and showed a clear separation between the wild variety *C. annuum* AC 1979 (no. 19) and the land races and breeders’ varieties in this group. Cluster B-2 contained two sub-groups, one consisting of the *C. annuum*-spII and the second one separating the *C. chinense*, *C. frutescens*, and *C. baccatum* accessions, conform the PCA results. Accession no. 2, *C. chinense* I1 PI 281428, which in the PCA plot was located in close proximity to the *C. annuum*-spII group (Fig. 2a), was biochemically different from the other *C. chinense* accessions and forms an outlier in cluster B2.

HCA of the set of 331 semi-polar metabolites revealed the presence of several metabolite groups, arbitrarily denoted A to H, characterised by their specific expression pattern across the 32 pepper accessions (Fig. 3). By using different metabolite databases, in combination with accurate mass MS^n^ fragmentation experiments, we could putatively identify in total 88 out of the 331 semi-polar metabolites (Supplemental Table S1). These metabolites belonged to a number of compound classes, such as flavonoids, phenylpropanoids, phenolics, acyclic diterpenoids, branched chain amino acid derivatives and fatty acid derivatives. Structurally related metabolites derived from the same metabolic pathway clustered together, as shown previously for volatiles in tomato (Tikunov et al. 2005) and pepper (Eggink et al. 2011). Compared to the three other species, all *C. annuum* accessions accumulated relatively high levels of the acyclic diterpenoid phytatetraene, which was present in the form of different conjugated derivatives (groups D, E and F). These diterpenoid glycosides are also called capsianosides (De Marino et al. 2006; Izumitani et al. 1990; Lee et al. 2009) and currently receive increasing interest for their presumed antioxidant (Shin et al. 2012) and pharmaceutical activities (Hashimoto et al. 1997). The difference between *C. annuum*-spI (cluster B-1) and *C.annuum*-spII (part of cluster B-2) was mainly due to variation in the decoration of capsianosides, as shown in metabolite groups D and E versus F, respectively. The *C. baccatum* group could be distinguished from all other genotypes due relatively high levels of several phenolic and phenylpropanoid glycosides, i.e. several coumaroyl, feruloyl and benzyl glycosides. Interestingly, three of these compounds had a sulfate conjugation as well. In addition, the *C. baccatum* accessions contained several unique double charged compounds which we have not been able to annotate so far.

A large proportion of the identified metabolites consisted of flavonoid glycosides. These represented different intermediates in the biosynthetic pathway leading from chalcones (naringenin chalcone), through flavanones to the major pepper flavonoids belonging to both flavones (luteolin and apigenin) and flavonols (quercetin and kaempferol). The abundance of specific flavonoid glycosides appeared to be, at least partly, responsible for the separation of *C. chinense* and *C. frutescens* from the other species (group B and C). Other flavonoid glycosides showed accumulation patterns which were subspecies or genotype-specific rather than species-specific (Group G and H). This was most obvious for *C. annuum* Long Sweet (no. 12), which contained strongly elevated levels of various flavanone, flavone and flavonol glycosides. This accession was previously shown to be a high-flavonoid pepper accession (Wahyuni et al. 2011). Unfortunately, it was not possible to see a species- or genotype-specific pattern in the flavonoid conjugation suggestive of the presence of specific modifying enzymes in certain genotypes or species groups.

Capsaicinoids were generally not detected in the LC–MS set-up used. However, we could detect one capsaicin analogue (Fig. 3; group B). The levels of this compound showed a very high correlation with the total capsaicinoid content in pericarp (*R* ^2^ = 0.98; Table 2), which was measured previously (Wahyuni et al. 2011). Other metabolites present in the same cluster showed a good correlation with capsaicinoid content as well (Table 2) and, interestingly, those metabolites for which we had a putative annotation all derived from the phenylpropanoid and the branched chain fatty acid pathway (consisting of methyl-branched amino acid degradation and fatty acid synthesis modules), both of which also serve to provide the immediate precursors for capsaicin biosynthesis (Mazourek et al. 2009).

**Table 2:** Pearson correlation of volatile and semi-polar metabolites with total capsaicinoid levels in pericarp, placenta and seeds and with the capsaicin analogue detected by LCMS

Type	Ret(min)	Mass	RI	Putative identity	HCA cluster	Correlation to capsaicin pericarp	Correlation to capsaicinseed and placenta	Correlation to capsaicin analog LCMS
Volatile	38.7	55	1657.5	9-Tetradecenal, (Z)-	A	0.90	0.53	0.88
	27.3	70	1253.7	Heptyl isobutanoate	A	0.86	0.41	0.82
	29.9	82	1332.7	Hexanoic acid, 3-hexenyl ester, (Z)-	A	0.85	0.57	0.79
	25.0	71	1187.3	Butanoic acid, hexyl ester	A	0.84	0.59	0.83
	23.8	56	1153.4	Hexyl 2-methylpropanoate	A	0.82	0.66	0.82
	38.8	41	1664	Oxacyclotetradecan-2-one	A	0.81	0.71	0.81
	21.7	71	1093.6	Butanoic acid, pentyl ester	A	0.81	0.36	0.79
	38.8	55	1662	Oxacyclotetradecan-2-one	A	0.80	0.72	0.81
	24.9	67	1183	cis-3-Hexenyl Butyrate	A	0.79	0.36	0.75
	23.3	103	1138	Pentyl 2-methylbutanoate	A	0.79	0.73	0.81
	27.0	56	1244.7	Hexyl *n*-valerate	A	0.78	0.70	0.78
	20.2	43	1051.4	Pentylisobutyrate	A	0.78	0.59	0.81
	28.1	56	1278.6	Hexyl *n*-valerate	A	0.78	0.58	0.78
	22.0	70	1101.7	Isopentyl 2-methylbutanoate	A	0.78	0.71	0.79
	16.7	43	956.08	3-Heptanone, 5-methyl-	A	0.77	0.29	0.67
	23.7	68	1148.3	3-Methyl-3-butenyl 3-methylbutanoate	A	0.76	0.71	0.79
	39.4	43	1683.6	13-Tetradecanolide	A	0.76	0.63	0.77
	23.7	70	1150.6	iso-Amyl isovalerate	A	0.75	0.58	0.75
	33.8	55	1466.5	1-Pentadecene	A	0.74	0.69	0.78
	35.6	56	1530.8	1-Tridecanol	A	0.74	0.66	0.74
	33.8	43	1468.2	*n*-Heptyl hexanoate	A	0.73	0.65	0.76
	18.8	43	1014.9	3-Methyl-1-butanol, 2-methylpropanoate	A	0.71	0.60	0.72
	38.5	43	1647.5	Hexadecane, 2-methyl-	A	0.70	0.47	0.68
	30.0	117	1337.7	4-methylpentyl 4-methylpentanoate	A	0.69	0.72	0.71
	28.0	82	1273.4	3-Hexen-1-ol valerate, (Z)-	A	0.69	0.47	0.61
	25.8	70	1209.7	Pentanoic acid, 4-methyl-, pentyl ester	A	0.68	0.66	0.66
	23.6	43	1145.6	Hexyl 2-methylpropanoate	A	0.67	0.71	0.70
	23.4	43	1141.7	*n*-Amyl isovalerate	A	0.66	0.76	0.70
	22.4	43	1111.8	Hexyl 2-methylpropanoate	A	0.65	0.73	0.68
	32.8	69	1430.7	alpha-Ionone	A	0.64	0.71	0.68
	22.1	70	1106.3	Butanoic acid, 3-methyl-, 3-methylbutyl ester	A	0.63	0.72	0.63
	20.4	43	1057.5	Pentyl 2-methylpropanoate	A	0.62	0.73	0.68
	25.4	56	1196.7	4-methylpentyl 3-methylbutanoate	A	0.61	0.76	0.64
	35.6	71	1531.7	gamma-Macrocarpene, (E)-	A	0.60	0.78	0.64
	12.2	56	829.52	1-Pentanol, 4-methyl-	A	0.60	0.86	0.64
	26.9	70	1242.4	Isopentyl hexanoate	A	0.59	0.38	0.60
	28.9	70	1301	4-Methyl-1-hexanol 2-methylbutanoate	A	0.58	0.76	0.60
	34.8	91	1499.6	gamma-Humulene	A	0.58	0.69	0.65
	25.9	69	1212.1	à-Citronellol	A	0.57	0.75	0.62
	34.8	93	1502.9	gamma-Humulene	A	0.56	0.67	0.63
	31.0	43	1368.9	alpha-Longipinene	A	0.53	0.65	0.59
	31.7	91	1392	Benzyl 3-methylbutanoate	A	0.51	0.75	0.51
	26.5	67	1230.6	Hexyl 2-methylbutanoate	A	0.48	0.68	0.52
	26.8	43	1238.1	Heptyl isobutanoate	A	0.48	0.50	0.52
	34.5	177	1490.7	beta-Ionone	A	0.47	0.64	0.51
	26.7	56	1234.2	Hexyl 3-methylbutanoate	A	0.47	0.68	0.51
	29.1	73	1308.8	Nonanoic acid	A	0.46	0.72	0.50
	31.3	82	1380	Hexenyl (3Z)-hexenoate, (3Z)-	A	0.45	0.56	0.48
	13.4	56	863.91	1-Hexanol	A	0.44	0.75	0.47
Semi-polar metabolites	39.5	318.17		capsaicin/capsaicin analogue	B	0.98	0.55	1.00
	17.9	395.19		Hexanol–pentose–hexose	B	0.87	0.33	0.87
	21.6	665.17		Chrysoeriol diglucose	B	0.76	0.28	0.66
	19.2	651.16		Luteolin-*O*-acetyl-diglucose	B	0.73	0.27	0.63
	13.9	903.24		Quercetin-dihexose-deoxyhexose-pentose	B	0.65	0.29	0.62
	12.6	447.15		Benzyl alcohol-hexose-pentose + FA	B	0.63	0.21	0.67
	18.4	725.19		3,4′,5,7-Tetrahydroxyflavone; 3-*O*-[Rhamnosyl-(1->?)-galactoside], 7-*O*-arabinoside	B	0.58	0.22	0.56
	13.3	337.09		Coumaroylquinic acid	B	0.57	0.12	0.60
	16.0	425.2		1-Hexanol;* O*-[?-d-Glucopyranosyl-(1?2)-?-d-glucopyranoside]	B	0.53	0.14	0.59

**Fig. 4:**
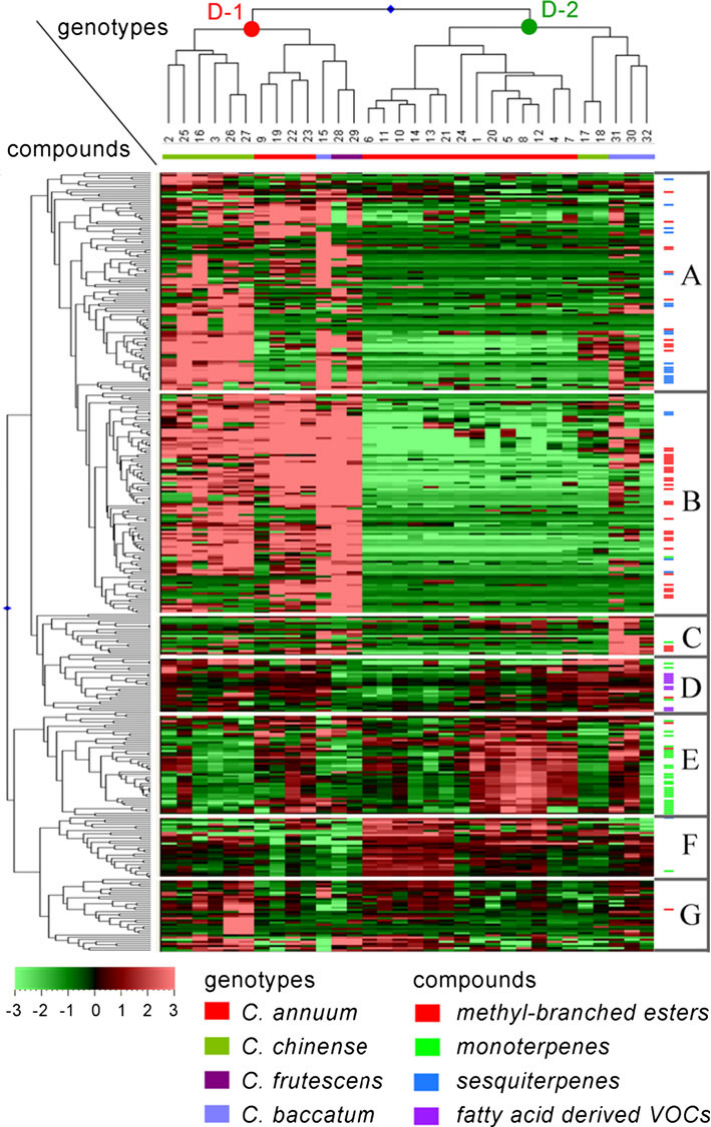
Heat map of 347 volatile metabolites in 32 pepper accessions. A *color-coded* matrix represents the mean values of the metabolite intensity in two biological replicates of pepper accessions, which has been log2 transformed and mean-centered. The *alphabets* (*A–G*) represent metabolite clusters. Characteristics of the underlying metabolites are presented in the Supplemental Table S2. The numbers below the dendrogram correspond to the accession numbers in the first column of Table 1

*Ret* retention time (minutes), *Mass* nominal mass (in case of volatiles) or exact mass (in case of semi-polar metabolites), *RI* retention index, *HCA cluster* refers to the cluster in the HCA for volatiles (Fig. 3) or semi-polar metabolites (Fig. 4). Only metabolites with a putative identity are shown. Total capsaicinoids used in the correlation analysis was measured previously (Wahyuni et al. 2011)

Metabolite cluster C consists of compounds with relatively high abundance in accession cluster B2, consisting of *C. annuum*-spII, *C. chinense*, *C. frutescens* and *C. baccatum*. Apart from three flavonoids, most metabolites within this cluster could not be identified so far.

#### Volatile metabolites

Headspace SPME–GC–MS chromatograms of ripe fruit pericarp samples from the 32 pepper accessions were subjected to full mass spectral alignment using MetAlign software, followed by grouping of the resulting 13,833 ion fragments into 408 putative volatile metabolites, using MSClust software. Of those 408 metabolites, 347 showed differential expression patterns based on ANOVA (*p* < 0.001) and were selected for further analysis. These 347 volatiles were putatively identified on the basis of both their mass spectrum and retention index, and are listed in Supplementary Table S2. In total 194 volatiles had a NIST-match factor above 600 and a retention index deviation of less than 50 units, and their putative identification was therefore considered as reliable. PCA analysis based on 347 volatiles (Fig. 2b) revealed that the separation into species groups was not as clear as observed for both the genetic data (Fig. 1) and the semi-polar metabolites (Fig. 2a). In fact, PC1, which explained 37.7 % of the total variation, differentiated the species groups according to pungency, as previously determined by measuring total capsaicinoid levels (Wahyuni et al. 2011). Accessions of *C. annuum* were clustered into two groups, denoted as *C. annuum*-vocI and *C. annuum*-vocII. *C. annuum*-vocI consisted of three mild pungent accessions: *C. anuum* Bisbas (no. 9), Chili Serrano (no. 22) and Chili de Arbol (no. 23), and one high pungent accession: *C. annuum* AC1979 (no. 19). The second group, *C. annuum*-vocII, contained the remaining *C. annuum* accessions, consisting of seven non-pungent accessions and seven low pungent accessions. In addition, PC1 also differentiated the *C. chinense* accessions into two groups based on pungency. Group one, denoted as *C. chinense*-vocI, consisted of six *C. chinense* accessions with a mild level of pungency, whereas *C. chinense*-vocII contained two low pungent accessions: *C. chinense* No. 4661 (no. 17) and No. 4661 Selection (no. 18). Also the four *C. baccatum* accessions were differentiated based on pungency: the high pungent *C. baccatum var. baccatum* (no. 15) was clearly separated from the three low/mild pungent accessions of *C. baccatum var. pendulum* and plotted close to the two high pungent accessions of *C. frutescens*.

**Fig. 5:**
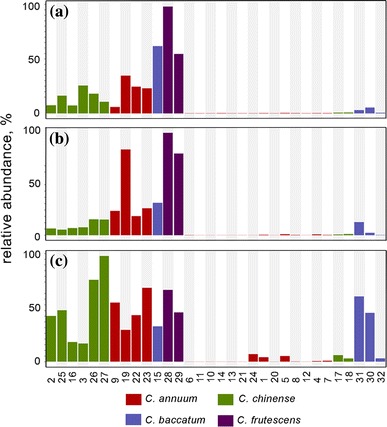
*Bar-plots* representing relative abundance of metabolites in 32 pepper accessions: **a** total methyl-branched esters in pericarp, **b** total capsaicinoids in pericarp and **c** total capsaicinoids in combined placenta and seeds. Total capsaicinoids in pericarp and placenta and seeds were based on Wahyuni et al. (2011). The *numbers* below the *bar plots* correspond to the accession numbers in the first column of Table 1

HCA revealed two main accession clusters, denoted as D-1 and D-2, (Fig. 4). This separation was driven by a large set of volatiles which were much more abundant in fruits of accession cluster D-1 compared to D-2. We arbitrary divided the volatiles characteristic for cluster D-1 into two groups A and B based on their accumulation pattern (Fig. 4). On average the similarity distances between volatiles of group B were the shortest over the whole dendrogram, indicating a very high pattern similarity across the accessions. A major portion of this volatile cluster consisted of various methyl-branched esters, which are products of the branched chain amino acid degradation pathway. Some of these compounds could also be observed in the neighbouring cluster A together with sesquiterpenes, which were clustered in this group. In summary, methyl-branched esters and sesquiterpenes were much more abundant in fruits of accession cluster D-1 compared to D-2. Interestingly, these two accession clusters correspond to the division that could be made based on fruit pungency, which was also apparent in the PCA results. Cluster D-1 was comprised of accessions with mild and high levels of pungency, corresponding to *C. annuum*-vocI, *C. chinense*-vocI, *C. frutescens* and *C. baccatum var. baccatum*. Cluster D-2 contained the low and non pungent accessions and consisted of three sub-clusters containing *C. annuum*-vocII, *C. chinense*-vocII and *C. baccatum var. pendulum*. The total amount of methyl-branched esters, in general, fits well with the quantitative patterns of capsaicinoids in both pericarp (*R* ^2^ = 0.9) and placenta (*R* ^2^ = 0.7) across all 32 accessions, as previously determined by Wahyuni et al. (2011), but detailed analysis of the pungent group shows that accumulation of these volatiles fit the capsaicinoid accumulation in pericarp better than capsaicinoid amounts found in placenta (Fig. 5; Table 2). The apparent division of genotypes based on pungency likely reflects the activity of the branched-chain amino-acid degradation pathway, which not only leads to the production of methyl-branched esters, but also provides precursors for the production of the acyl moiety of capsaicinoids.

Monoterpene volatiles, clustered in compound group E, drove further differentiation of *C. annuum*-vocII into six sweet breeders’ varieties (accession no. 6, 11, 10, 14, 13 and 21) and the low pungent, pointed *C. annuum* accessions (accession no. 24, 1, 20, 5, 8, 12, 4 and 7), which showed a significantly higher emission of monoterpenes. In addition, *C. chinense* accessions generally showed a low emission of monoterpenes. Another group of volatiles showing biochemically driven compound clustering is group D, in which several volatiles originating from the lipoxygenase pathway showed very similar intensity patterns across the accessions and were on average low in the six sweet breeder’s *C. annuum* accessions mentioned above. Group G did not reveal a clear biochemically driven compound clustering as it consisted of volatiles of various biochemical origin.

In general, methyl-branched esters and sesquiterpenes appeared to be the most differentiating compounds between the accessions analysed. Their quantitative patterns show a high similarity to the pungency pattern of the accessions suggesting that these volatiles and capsaicinoids may have common factors which control their accumulation in pepper fruit.

## Discussion

The dendrogram produced using AFLP markers revealed the phylogenetic relationship between the four *Capsicum* species used in this study. The phylogenetic relationships fit well with the geographic dispersion of the species and the common ancestor of the *C. annuum*–*C. chinense*–*C. frutescens* species complex, which diverged from *C. baccatum* at an early stage during evolution (Basu and De 2003; Djian-Caporilano et al. 2007; Eshbaugh 1970). This is also in line with results from previous studies in which different *Capsicum* germplasm was compared (Ince et al. 2010; Kochieva et al. 2004; Toquica et al. 2003). Further differentiation can be seen within the *C. annuum* group, in which bell pepper breeding lines and pointed peppers formed separate groups. This molecular differentiation most likely reflects breeders’ efforts to develop and genetically select for specific pepper fruit types.

The overall composition of semi-polar metabolites was strongly determined by the species group, indicating that genetic differences between species are reflected in metabolic differences. This may be due to differences in the regulation of metabolic pathways, differences in the activity of key enzymes determining the flux through the pathway, or the activity or substrate specificity of specific modifying enzymes. The importance of the latter enzyme group was illustrated by the large, species-specific, variation in the “decoration” of flavonoids and capsianosides. In contrast to the AFLP data, semi-polar metabolites differentiated the *C. annuum* group from *C*. *chinense*–*C*. *frutescens*–*C*. *baccatum*. This differentiation may be due to the domestication and selection history of the accessions studied. Indeed, *C. annuum* accessions have been most commonly used for breeding purposes and therefore may have been exposed to the strongest selection history, including selection for desirable traits during breeding. Selection on specific mutations will have a marginal impact on the total genetic make-up of the species group, but may strongly affect the expression of genes thereby altering specific metabolic pathways, as shown for tomato ripening (Kovács et al. 2009), or high pigment (Bino et al. 2005; Levin et al. 2006) mutants. In pepper, this is clearly demonstrated by comparing the position of the wild variety *C. annuum* AC 1979 (no. 19) relative to those of the cultivated *C. annuum* accessions in the phylogenetic tree based on AFLP markers (Fig. 1) with its position in the HCA based on semi-polar metabolites (Fig. 3).

Unlike the overall metabolite profiles, targeted analyses of a specific set of metabolites, such as carotenoids, capsaicinoids and vitamins C and E, revealed a large variation between individual accessions rather than between species groups (Wahyuni et al. 2011). Variation in these health-related metabolites may have resulted from breeding efforts aimed at selecting for consumer-driven attributes such as carotenoids for fruit color and capsaicinoid levels to enhance or avoid pungency, rather than from differences between species.

In case of volatiles, the accessions were primarily clustered based on pungency rather than on species (Fig. 2b). Identification of the volatiles driving this contrast revealed a relatively high abundance of sesquiterpenes and methyl-branched fatty acid esters in the pericarp of pungent accessions. The total amount of methyl-branched fatty acid esters showed a strong correlation with the total amount of capsaicinoids in the pericarp and, to a lesser extent, to capsaicinoids in the placenta (Fig. 5) and the same holds for the individual volatiles in metabolite cluster A of the HCA, including four sesquiterpenes (Table 2). In this respect we also observed a close clustering of methyl-branched esters with the capsaicin analog in a HCA based on a combined dataset of both semi-polar and volatile metabolites (Supplemental Fig. 1; Table S3). However, there is no obvious relation between the metabolic pathways leading to sesquiterpenes and the pungent capsaicinoids, suggesting that this correlation is most likely due to population structure in this germplasm collection rather than to a causal relationship. This can, however, only be addressed by the use of segregating populations. We can not exclude that population structure may also account for the correlation of methyl-branched esters with pungency. However, methyl-branched fatty acid esters are derived from the catabolism of branched-chain amino acids, such as valine, leucine and isoleucine, which also serve as direct precursors for the acyl branch of capsaicinoid biosynthesis (Mazourek et al. 2009). Hence, methyl-branched fatty acid esters may reflect the activity of the capsaicinoid pathway. The primary site of capsaicinoid production is the placenta, from where capsaicinoids are transported into the apoplast and stored in so-called ‘blisters’, or towards other tissues or organs, such as the fruit pericarp, stems and leaves (Broderick and Cooke 2009; Estrada et al. 2000; Kim et al. 2011; Stewart et al. 2007). The strong correlation of methyl-branched fatty acid ester levels with capsaicinoid levels in the pericarp suggests that these methyl-branched esters are also synthesized in the placenta from where they are subsequently transported to the pericarp. Indeed, Moreno et al. (2012) recently showed a 12–48-fold higher accumulation of methyl-branched esters in the placenta compared to the pericarp.

In pepper, pungency is primarily determined by the *Pun1* gene, which encodes capsaicin synthase, the final step in capsaicinoid biosynthesis. This putative acyltransferase is responsible for the formation of capsaicinoids through transfer of the acyl moiety of the capsaicin pathway to vanillin-amide, the product of the aromatic branch of the capsaicin pathway (Mazourek et al. 2009; Stewart et al. 2005). Loss of function mutations in this gene lead to the absence of capsaicinoids and a lack of pungency. Quantitative differences in pungency likely reflect the activity through the aromatic and acyl branches of the capsaicin pathway and several QTLs affecting pungency have been described (Ben-Chaim et al. 2006; Blum et al. 2003; Mazourek et al. 2009). Most of the current sweet pepper breeding germplasm has a loss of function mutation in the *pun1* gene (Mazourek et al. 2009). It is evident that these accessions not only lack capsaicinoids, but are also low in methyl-branched fatty acid esters, suggesting that the entire pathway leading to capsaicinoids is down-regulated in these accessions. There are several possible explanations for this observation. Firstly, it is possible that the *Pun1* locus or capsaicinoids, directly or indirectly, regulate the flux through the entire upstream biochemical pathway, as suggested by increased and co-regulated expression of several capsaicin pathway genes in *Pun1* compared to *pun1* genotypes (Stewart et al. 2005; Stewart et al. 2007). Secondly, we cannot exclude that the capsaicin synthase enzyme has a broad substrate preference and also functions as an ester-forming enzyme required for the esterification of methyl-branched fatty acid esters (Stewart et al. 2005). Thirdly, it is possible that breeding efforts selecting for sweet bell peppers not only targeted the *pun1* mutation, but also selected for genotypes with a low activity of the upstream branches of the capsaicin pathway, which may be due to mutations in one or more genes encoding enzymes or regulatory factors of this pathway. The latter option would make it possible to introgress the pungent aroma into a non-pungent pepper background.

(Sub)species-specific clustering of volatiles was mainly due to qualitative and quantitative differences in the accumulation of metabolites derived from the major pathways leading to volatile production: aromatic and branched chain amino acid catabolism, fatty acid degradation, mono- and sesquiterpene biosynthesis and, to a lesser extent, carotenoid degradation, reflecting variation in the activities and substrate specificities of the corresponding enzymes. Although we did not conduct any sensorial analyses on these accessions, it may be expected that the observed metabolic differences form the basis for species-specific differences in aroma and opens up possibilities to breed for novel aromas in cultivated pepper backgrounds. Rodríguez-Burruezo et al. (2010) determined the volatile composition in ripe fruits of 16 *Capsicum* accessions from the *annuum*–*chinense*–*frutescens* complex and combined their metabolite analysis with taste panel data and sniffing port analyses. They concluded that the diversity in aromas found in their accessions was due to variation in the levels of at least 23 odour-contributing volatiles. Of those, 16 could also be detected in our data set (Table 3). In agreement with Rodríguez-Burruezo et al. (2010), we found that pungent accessions contain the highest levels of aroma-contributing volatiles, such as the sweet, fruity methyl-branched esters hexyl-2/3-methylbutanoate, 4-methylpentyl-3-methylbutanoate and 4-methylpentyl-4-methylpentanoate, the fatty acid derived aldehydes nonenal and nonedienal, which give rise to a green cucumber-like odour impression, as well as the fruity, floral carotenoid-derived volatiles alpha- and beta-ionone (Fig. 4, clusters A and B). The characteristic green bell pepper volatiles pyrazine and 2-heptanethiol were detected at varying levels in all genotypes analysed, irrespective of pungency level or species. Pointed *C. annuum* accessions were relatively rich in the monoterpenes alpha-pinene (pine, wood-like), linalool (citrus, fruity, floral) and 1,8-cineole (eucalyptus-like), as well as the hydrocarbon ectocarpene which has a green, sweet odor description (Fig. 4, cluster E). Finally, we observed that the important fatty-acid derived volatile hexanal was most abundant in all *C. baccatum* accessions analysed (Fig. 4, cluster D).

**Table 3:** Relative abundance of 16 odour-contributing volatiles in 32 pepper accessions

Odour descriptions are derived from Rodríguez-Burruezo et al*.* (2010). Genotypes are represented in the same order as in the HCA (Fig. 4). Values represent log_2_ values of mass peak intensities determined for each volatile in the set of 32 pepper accessions. Colours represent relative intensities for each volatile from dark green (low intensity) to dark red (high intensity)

## Conclusions

The results of this study clearly demonstrate that there is a large metabolic variation present in the pepper germplasm collection analysed. Species-driven metabolic differences are the major determinants of the variation in semi-polar metabolites, whereas pungency was the main driver responsible for the variation in aroma volatiles. The levels of the metabolites analysed varied greatly among fruits of different accessions, demonstrating the potential of the current germplasm collection for genetic improvement of metabolic traits. In addition to promising sources of health-related flavonoids and capsianosides, we identified accessions with high levels of several established flavour-related volatiles, such as methyl-branched fatty acid esters, fatty acid derived volatiles, such as hexanal, nonenal and nonedienal, and monoterpenes. These accessions are potential candidates for breeding programs aimed at developing new pepper cultivars with improved flavour and other consumer quality characteristics. Our results also indicate the value to explore the metabolic variation with different analytical platforms and to couple metabolomics with genetic analysis as a strategy to target crop breeding programs to phenotypic diversity for important quality traits.

## Electronic supplementary material

Below is the link to the electronic supplementary material.

Supplementary material 2 (XLSX 162 kb)

Supplementary material 3 (XLSX 171 kb)

Supplementary material 4 (XLSX 312 kb)

Supplementary material 1 (PPTX 177 kb)
