# Supplementary material for: Metabolomics and molecular marker analysis to explore pepper (Capsicum sp.) biodiversity
Source: Metabolomics. 2012 Jun 2;9(1):130–44. doi: 10.1007/s11306-012-0432-6 (PMC3548101; doi:10.1007/s11306-012-0432-6)
Supplement: Supplementary file 4 — Supplementary material 1 (PPTX 177 kb) [file 11306_2012_432_MOESM4_ESM.pptx]

## Slide 1
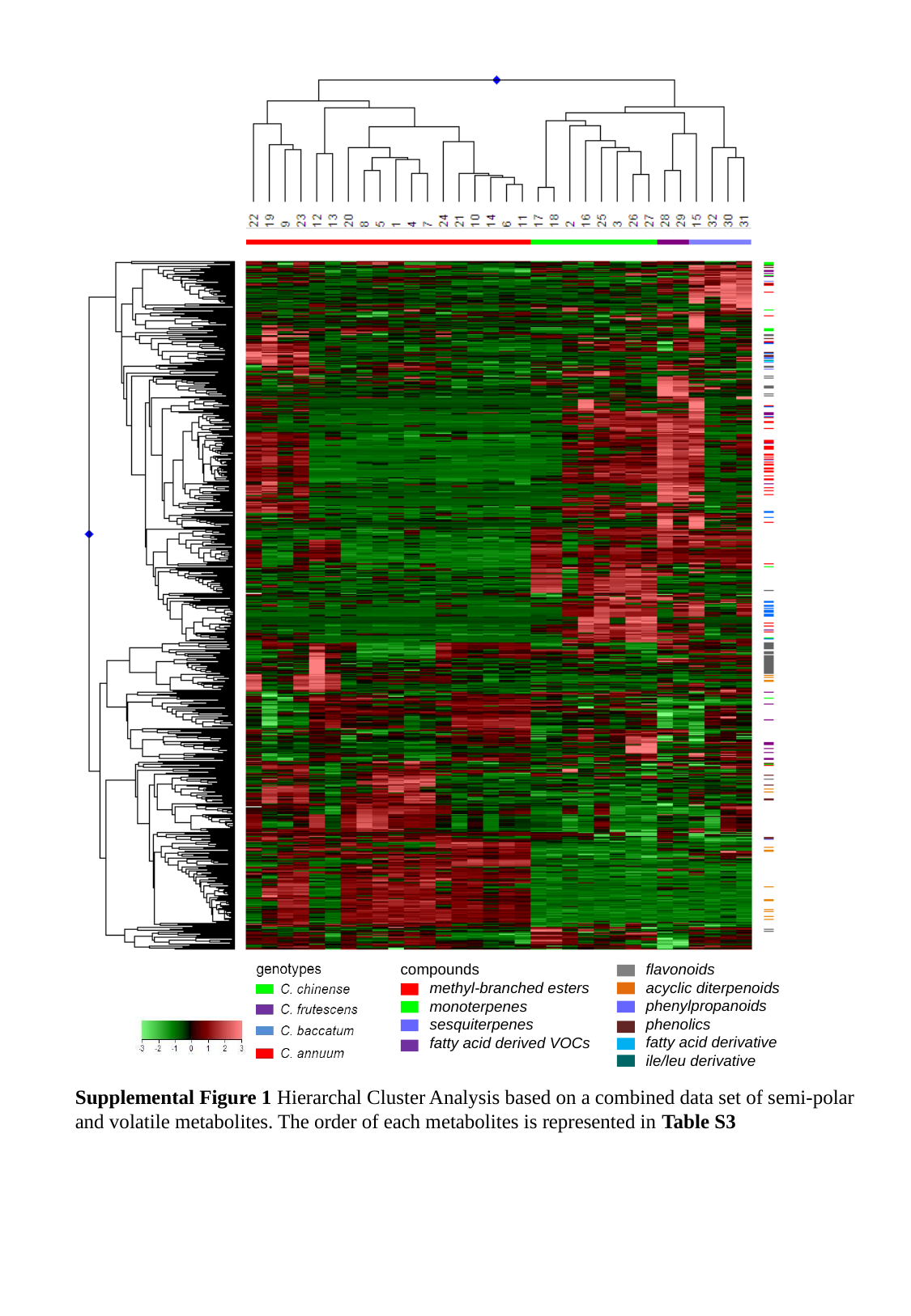

flavonoids
acyclic diterpenoids
phenylpropanoids
phenolics
fatty acid derivative
ile/leu derivative
compounds
methyl-branched esters
monoterpenes
sesquiterpenes
fatty acid derived VOCs
Supplemental Figure 1 Hierarchal Cluster Analysis based on a combined data set of semi-polar and volatile metabolites. The order of each metabolites is represented in Table S3
